# Expression of osteoprotegerin and osteoprotegerin ligand in giant cell tumor of bone and its clinical significance

**DOI:** 10.3892/ol.2013.1199

**Published:** 2013-02-19

**Authors:** XIUCHUN YU, WEIQING KONG, KAI ZHENG

**Affiliations:** Orthopedic Department, The General Hospital of Jinan Military Commanding Region, Jinan, Shandong 250031, P.R. China

**Keywords:** giant cell tumor of bone, osteoprotegerin, osteoprotegerin ligand, immunohistochemistry

## Abstract

In this study, we used a substance P (SP) immunohistochemical method to analyze the expression localization of osteoprotegerin (OPG) and osteoprotegerin ligand (OPGL) in giant cell tumor (GCT) of the bone, and to detect the clinical significance of their expression. The data showed that the positive expression rate of OPG in the multinucleated giant cells (MGCs) and stromal cells (STCs) of GCT was 80.65 and 74.19%, respectively. The positive expression rate of OPG in MGCs was correlated with age and prognosis (P<0.05), but not in STCs. The strength of positive OPG expression in MGCs and STCs was negatively correlated with prognosis (rs=−0.397, P<0.05; rs=−0.390, P<0.05, respectively). The positive expression rate of OPGL in the MGCs and STCs was 41.94 and 67.74%, respectively. The positive expression rate of OPGL in the MGCs was correlated with age and prognosis (P<0.05); the strength of OPGL expression in MGCs was positively correlated with Campanicci’s grade and recurrence. Additionally, the positive expression rate of OPGL in STCs was correlated with age and Jaffe’s grade (P<0.05). The strength of OPGL expression in STCs was negatively correlated with Jaffe’s grade (rs=−0.534, P<0.05). In conclusion, OPG and OPGL are expressed in MGCs and STCs in GCT of the bone. The invasion of tumor cells was positively correlated with OPGL in MGCs, which confirmed that MGCs participate in the process of osteolytic destruction of GCT of bone.

## Introduction

Giant cell tumor (GCT) of the bone is an osteolytic tumor that is potentially malignant or is between benign and malignant, and is characterized by local invasive destruction of bone (1) and a high post-curettage recurrence rate (2). The mechanisms underlying the osteolytic destruction of bone have been demonstrated (2,3). Tumor cells directly or indirectly stimulate the differentiation and maturation of osteoclasts and promote bone resorption. However, multiple growth factors released from the bone matrix promote tumor cell seeding in the bone tissue, which in turn exacerbates the invasive growth of tumor cells in the bone tissue. During this process, the activation of osteoclasts is the key step. It has been suggested that the osteoprotegerin (OPG)/receptor activator of NF-κB (RANK)/RANK ligand (RANKL) system is vital for the osteolytic destruction of bones mediated by osteoclasts (3,4). Hence, the involvement of the OPG/RANK/RANKL system in the osteolytic features of GCT has received increasing attention from orthopedists. In the present study, we investigated the expression of OPG and OPG ligand (OPGL) in GCT by immunohistochemical analysis, to explore the correlation between their expression and tumor invasiveness. Our study provides molecular biological evidence for the clinical application of bisphosphonate drugs in the treatment of GCT.

## Materials and methods

### 

#### Clinical data

The present study included 18 male and 13 female patients with an average age of 35.19 years (range, 13–78 years). The study was approved by the Ethics Committee of the Orthopedic Department, The General Hospital of Jinan Militray Commanding Region, Jinan, Shandong, China. Written informed consent was obtained from the patients and patient’s family. Of the 31 patients, there were 9 cases of distal femur tumor, 9 cases of proximal tibia, 4 cases of proximal femur, 3 cases of proximal humerus, 2 cases of iliac, 1 case of distal radius, 1 case of distal ulna, 1 case of pubis and 1 case of calcaneus. According to Jaffe’s classification criteria, there were 12 cases of class I, 17 cases of class II and 2 cases of class III tumors. Based on Campanicci’s radiological classification criteria, there were 7 cases of class I, 16 cases of class II and 8 cases of class III tumors. All patients were treated with tumor curettage and bone grafting surgeries performed by the same surgical group.

All 31 patients received follow-up surveys for an average duration of 76 months (range, 24–124 months). No metastasis was observed. Eight patients exhibited recurrence, and the second surgery included 1 case of tumor curettage and bone grafting, 4 cases of tumor resection and prosthesis replacement, 1 case of tumor resection and inactivated replantation,and 1 case of extended tumor resection. Following secondary surgery, the 8 patients received follow-up for an average duration of 50 months (range, 20–124 months), and no metastasis or recurrence was observed.

#### Immunohistochemical analysis of the expression of OPG and OPGL in GCT

Polyclonal rabbit anti-human antibodies specific for OPG and OPGL were purchased from Boshide (Wuhan, China). The substance P (SP; rabbit) immunohistochemistry kit was purchased from Zhongshanjingqiao Biotechnical Co., Ltd. (Beijing, China).

H&E-stained slices of the 31 GCT pathological specimens were reviewed and typical paraffin-embedded samples were selected for analysis. The 31 representative samples were used to prepare 3 paraffin sections with a thickness of 4 *μ*m for H&E staining and OPG/OPGL analysis. All methods were performed according to the manufacturer’s instructions. The slice with positive expression was used as the positive control. The negative control was established with the primary antibody replaced by phosphate-buffered saline (PBS).

**Figure 1:**
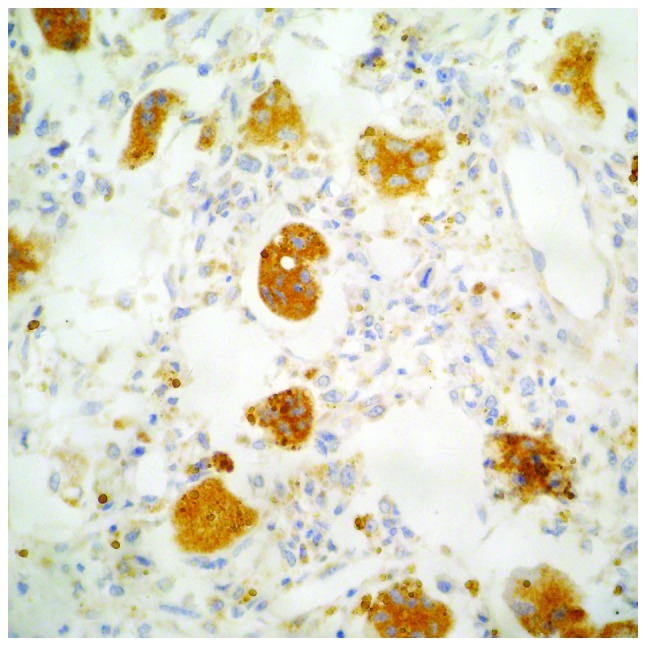
The expression of osteoprotegerin (OPG) (stained brown) is only evident in the multinucleated giant cells in giant cell tumor (GCT), and is located in the intracytoplasm (SP, ×400).

**Figure 2:**
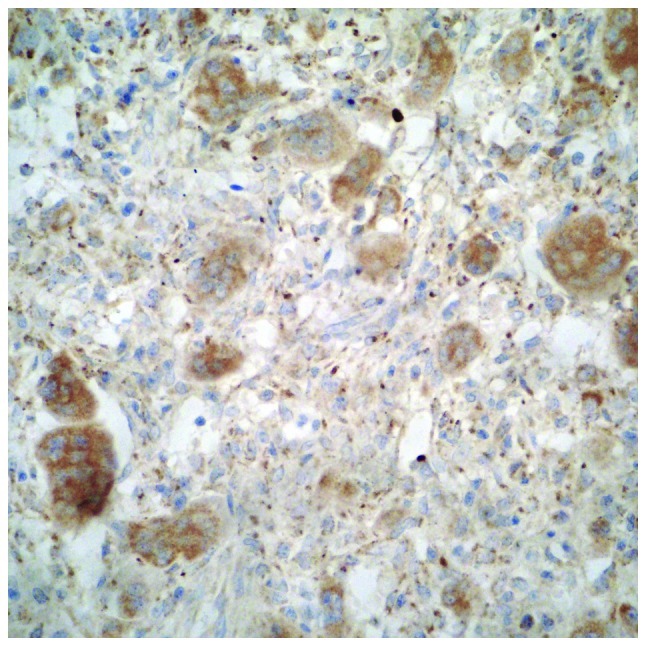
The expression of osteoprotegerin (OPG) is strongly positive in giant cell tumor (GCT), and is located in the multinucleated giant cells and stromal cells (SP, ×400).

**Figure 3:**
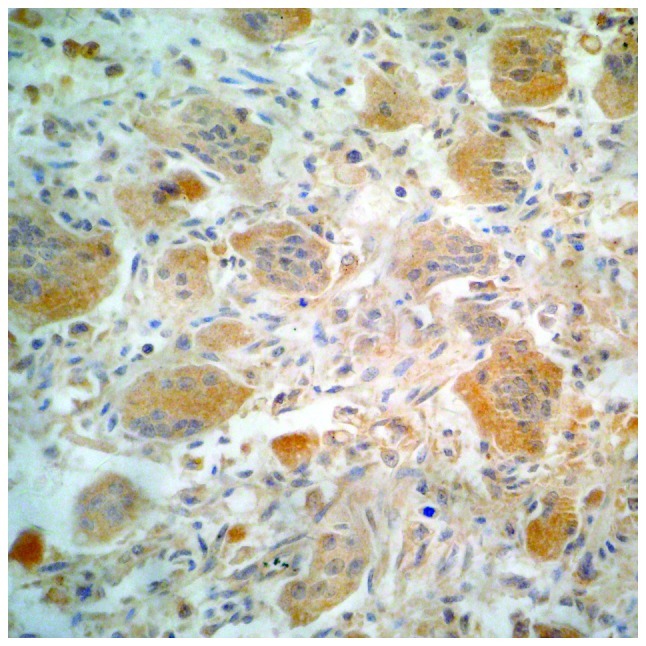
The expression of osteoprotegerin ligand (OPGL; stained jasmine) is only evident in the multinucleated giant cells in giant cell tumor (GCT), and is located in the intracytoplasm (SP, ×400).

**Figure 4:**
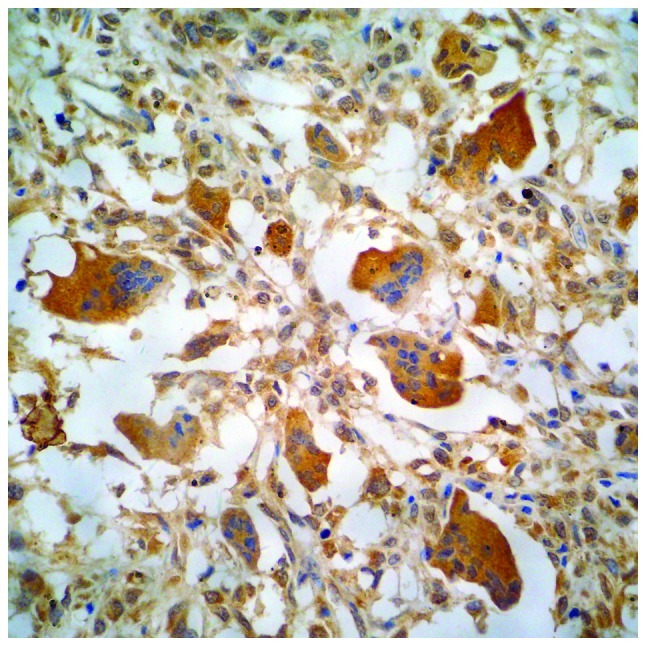
The expression of osteoprotegerin ligand (OPGL) is strongly positive in giant cell tumor (GCT), and is located in the multinucleated giant cells and stromal cells (SP, ×400).

**Figure 5:**
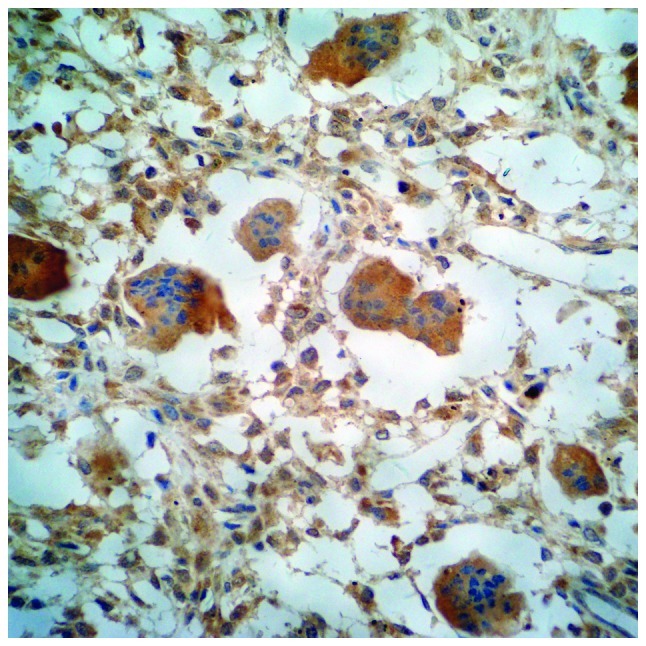
The expression of osteoprotegerin (OPG) is positive in giant cell tumor (GCT), and is located in the multinucleated giant cells and stromal cells (SP, ×400).

Both OPG and OPGL were expressed in stromal cells (STCs) and multinucleated giant cells (MGCs). Positive expression was determined by yellow, brown and tan particles on the plasma membrane or in the cytosol (Figs. 1–5). The slices were scored based on the integrated staining intensity and positive cell number. The slices with the highest score were selected for microscopic analysis. For each slice, 10 high magnification fields (10×40) were randomly selected to analyze 100 cells per field (1,000 tumor cells for 10 fields). The criteria used to determine the OPG and OPGL expression were described previously (5). The cells were scored based on the staining intensity of the plasma membrane and cytosol, and the percentage of stained cells. The cell was negative if the product of the two scores was ≤3, weakly positive if the product was between 4 and 6 (+), positive if the product was 7 or 8 (++), and strongly positive if the product was between 9 and 12 (+++).

#### Statistical analysis

The Wilcoxon rank-sum test, Kruskal-Wallis H test and Spearman’s correlation method were used to analyze the correlation of the OPG and OPGL expression levels with age, gender, tumor site, Jaffe’s class, Campanicci’s class and prognosis. All data analysis was performed using SPSS 17.0 software (SPSS, Inc., Chicago, IL, USA). P<0.05 was considered to indicate a statistically significant difference.

## Results

### Expression of OPG in the GCT

#### Correlation between OPG expression in the MGCs and clinical pathological characteristics

Of the 31 cases of GCT, 25 exhibited positive OPG expression in the MGCs, and 6 demonstrated negative expression. The OPG positive expression rate was 80.65%. Further analysis revealed that the expression of OPG in the MGCs was not correlated with gender, tumor site, Jaffe’s class or Campanicci’s class (P>0.05). The patients between 21 and 40 years demonstrated a positive rate of 94.74%, which was significantly higher than that of the other two age groups (P<0.05). Notably, the expression of OPG in the MGCs was correlated with recurrence (P<0.05; Table I).

#### Correlation between OPG expression in the STCs and clinical pathological characteristics

Of the 31 cases of GCT, 23 demonstrated positive OPG expression in the STCs, and 8 exhibited negative expression. The OPG positive expression rate was 74.19%. Further analysis revealed that the expression of OPG in the STCs was not correlated with gender, age, tumor site, Jaffe’s class, Campanicci’s class or prognosis (P>0.05; Table II).

#### Correlation between OPG expression in the GCT and clinical pathological characteristics

The Spearman’s analysis revealed that the expression of OPG in the MGCs and the STCs was not correlated with gender, age, tumor site, Jaffe’s class or Campanicci’s class (P>0.05); however, it was negatively correlated with prognosis (rs=−0.397 and P=0.027 for MGCs; rs=−0.390 and P=0.030 for STCs).

### Expression of OPGL in the GCT

#### Correlation between OPGL expression in the MGCs and clinical pathological characteristics

Of the 31 cases of GCT, 13 demonstrated positive OPGL expression in the MGCs, and 18 had negative expression. The OPG positive expression rate was 41.94%. Further analysis revealed that the expression of OPGL in the MGCs was not correlated with gender, tumor site, Jaffe’s class or Campanicci’s class (P>0.05). The patients <20 years demonstrated a positive rate of 100%, which was significantly higher than that of the other two age groups (P<0.05). Notably, the expression of OPGL in the MGCs was correlated with recurrence (P<0.05; Table III).

#### The correlation between OPGL expression in the STCs and clinical pathological characteristics

Of the 31 cases of GCT, 21 exhibited positive OPGL expression in the STCs, and 10 demonstrated negative expression. The OPG-positive rate was 67.74%. Further analysis revealed that the expression of OPGL in the STCs was not correlated with gender, tumor site, Campanicci’s class or prognosis (P>0.05). The patients between 21 and 40 years demonstrated a positive rate of 84.21%, which was significantly higher than that of the other two age groups (P<0.05). Importantly, the OPGL positive expression rate was significantly higher in the Jaffe’s class I group (91.67%) than in the Jaffe’s class II and III groups (P<0.05; Table IV).

#### Correlation between OPGL expression in the GCT and clinical pathological characteristics

The Spearman’s analysis revealed that the expression of OPGL in the MGCs was not correlated with gender, age, tumor site, Jaffe’s class or prognosis (P>0.05), but it was positively correlated with Campanicci’s class (rs= 0.377, P= 0.037). The expression of OPGL in the STCs was not correlated with gender, age, tumor site, Campanicci’s class or prognosis (P>0.05), but it was negatively correlated with Jaffe’s class (rs=−0.534, P=0.002).

## Discussion

GCT is a common type of primary bone tumor and accounts for 5–8% of the incidence of bone tumors. GCT commonly occurs in 20 to 50-year-old individuals, and is mostly focused on the metaphysis, particularly around the knee (∼65%). The invasion of the GCT is mainly due to the osteolytic destruction of the local bone. At present, the major surgical approaches for GCT treatment include intralesional excision and *en bloc* or wide resection. Intralesional excision has been used as the primary approach for the treatment of GCT, but the recurrence rate is as high as 20–50%. Wide excision is able to reduce the recurrence rate, but the frequent occurrence of long-term complications reduces the clinical efficacy. Although the development of bisphosphonate drugs has provided a potent method for retaining the joints of the patients and for improving the clinical efficacy, limited information is available regarding the mechanisms underlying the effects of these drugs. In the present study, we retrospectively analyzed 31 patients with complete clinical data for the past 10 years to provide scientific evidence for the clinical use of bisphosphonate drugs. We investigated the expression of OPG and OPGL in the GCTs using immunohistochemical analysis, to explore the correlation between their expression and the clinical characteristics of the tumor.

OPG is a soluble protein secreted by osteoblasts and bone marrow stromal cells. OPG is also a decoy receptor, with OPGL (also known as RANKL) as its ligand. By binding to the RANKL secreted by the bone marrow stromal cells, OPG blocks the interaction between RANKL and RANK, and subsequently acts to inhibit the differentiation of osteoclasts and the bone resorption activity of mature osteoclasts. In this way, OPG is able to induce the apoptosis of osteoclasts, reduce bone resorption and protect the bone. In addition, OPG is a member of the TNF receptor superfamily, and is capable of binding to TNF ligands.

RANKL is mainly expressed in osteoblasts and bone marrow stromal cells. RANKL binds to the plasma membrane, and is subsequently localized on the surface of osteoblasts and bone marrow stromal cells. The receptor of RANKL is RANK, which is usually localized to the surface of osteoclast precursors. In the presence of macrophage colony stimulating factor (M-CSF), when the osteoclast precursors and osteoblasts or bone marrow stromal cells come into contact with each other, RANKL binds to RANK on the surface of the osteoclasts and subsequently induces the activation and differentiation of the osteoclasts through the intracellular signaling pathway. RANKL can simultaneously enhance the activity of the mature osteoclasts and prevent osteoclast apoptosis. Therefore, bone destruction caused by RANKL-mediated osteoclast activation is necessary for the invasive growth of tumors.

The expression of OPG and OPGL in GCTs has been demonstrated. Guo *et al*(6) found that OPG is enriched in all types of GCT cells. Meng *et al*(7) found that the OPG protein is expressed in the MGCs and some STCs in GCTs. Hu *et al*(8) and Liu *et al*(9) both demonstrated that OPG is located in the MGCs and STCs of GCTs, indicating that a negative feedback mechanism exists in GCT and acts to inhibit osteoclast formation and bone resorption. However, this feedback is likely to be insufficient to counteract the effect of RANK. Hence, bone resorption may still occur in the GCT tissues, of which the clinical symptom is bone destruction.

Atkins *et al*(10) isolated STCs in the GCT, using RT-PCR assay, and detected the expression of RANKL mRNA. Huang *et al*(11) found that RANKL is mainly expressed in the STCs, using the fluorescence *in situ* hybridization assay. Hu *et al*(8) revealed that RANKL mRNA is enriched in GCTs, and the ratio of RANKL mRNA to GAPDH is greater compared with in normal bone tissues. Roux *et al*(12) demonstrated that RANKL is expressed in the STCs and confirmed that it is secreted by these cells, using immunohistochemical assay. Zhu *et al*(13) performed immunohistochemical analysis and found that RANKL is expressed in both the MGCs and STCs in GCTs. In Zhu *et al*, 23 of the 44 GCT cases had RANKL-positive STCs and 10 cases exhibited RANKL-positive MGCs, with a positive rate of 52 and 23%, respectively. Furthermore, the RANKL expression in the STCs was negatively correlated with Jaffe’s class, which was consistent with the morphological observation of reduced NGC number, further indicating that RANKL is a key molecule for the formation of MGCs.

In the present study, we found that OPG and OPGL were expressed in both the MGCs and the STCs in the GCTs. OPG exhibited a positive rate of 80.65% in the MGCs and 74.19% in the STCs. OPGL demonstrated a positive rate of 41.94% in the MGCs and 67.74% in the STCs, which were significantly different. Statistical analysis revealed that the positive rate and expression level of OPG and OPGL in the MGCs and the STCs were correlated with multiple clinical characteristics, which is consistent with previous studies. We also observed the following results:

Firstly, the positive rate and expression level of OPG and OPGL were different among the three age groups. The positive rate of OPG in the MGCs was significantly higher in the 21 to 40-year-old patient group (94.74%) than in the other two age groups (P<0.05). The positive rate of OPGL in the STCs was significantly higher in the 21 to 40-year-old patient group (84.21%) than in the other two age groups (P<0.05). The positive rate of OPGL in the MGCs was significantly higher in the ≤20-year-old patient group (100.00%) than in the other two age groups (P<0.05). These results indicated that the MGCs were the main cause of bone destruction during the course of the GCT before the age of 20 years, while the STCs were the key reason for the bone destruction between the ages of 21 and 40 years.

Secondly, the expression of OPG and OPGL in different types of cells in the GCT was able to affect the prognosis. The positive rate of OPG in the MGCs was significantly higher for the non-recurrence group (91.30%) than for the recurrence group (50.00%; P<0.05). The expression level of OPG in the MGCs and STCs was negatively correlated with prognosis (rs=−0.397, P= 0.027; rs=−0.390, P= 0.030, respectively). Notably, the higher the OPG expression level in the MGCs and STCs, the higher the recurrence probability observed. The positive rate of OPGL in the MGCs was significantly higher for the recurrence group than for the non-recurrence group (P<0.05), indicating that the expression of OPG in the MGCs may be used as an important indicator for the prognosis.

Thirdly, the expression of OPGL in different types of cells in the GCT was correlated with classification. The OPGL positive expression rate of the STCs was significantly higher in the Jaffe’s class I GCT (91.67%) than in the Jaffe’s class II and III GCT (P<0.05). In the MGCs, the expression level of OPGL was positively correlated with Campanicci’s classification (rs= 0.377, P= 0.037); whereas in the STCs, the expression level of OPGL was negatively correlated to the Jaffe’s classification (rs=−0.534, P=0.002). These results indicated that MGCs and STCs have different biological functions. Although it has been suggested that GCT is mainly composed of STCs and the MGCs are only responsive cells, the origin, properties and functions of MGCs remain unclear. It has been suggested that MGCs play the role of osteoclasts. Thompson *et al*(14) proposed that MGCs are foreign body giant cells. Liu *et al*(15), found that MGCs are functionally analogous to osteoclasts, using immunohistochemical staining and enzyme staining assays, and suggested that MGCs are a type of osteoclast-like multinucleated giant cell that has bone resorption activity. In the present study, our results further suggested that MGCs are likely to directly induce osteolytic bone destruction. Furthermore, RANKL was able to regulate the differentiation and maturation of the MGCs, to a certain extent, indicating that RANKL is closely associated with bone destruction. Our observation is consistent with that of Atkins *et al*, in that the osteolytic bone destruction of GCTs is mediated by the OPG/RANK/RANKL signaling system (16).

## Figures and Tables

**Table I:** Correlation between the expression of OPG in MGCs and clinical pathology.

Category	No. of patients	OPG expression in MGCs			Z or Hc	P-value
		Positive	Negative	Rate (%)		
Gender						
Male	18	15	3	83.33	0.439	0.661
Female	13	10	3	76.92		
Age (years)						
≤20	4	1	3	25.00	10.176	0.006
21–40	19	18	1	94.74		
>40	8	6	2	75.00		
Tumor site						
Surrounding knee joint	18	15	3	83.33	0.439	0.661
Other	13	10	3	76.92		
Jaffe’s grading						
I	12	10	2	83.33	0.702	0.704
II	17	13	4	76.47		
III	2	2	0	100.00		
Campanicci’s grading						
I	7	4	3	57.14	3.096	0.213
II	16	14	2	87.50		
III	8	7	1	87.50		
Prognosis						
Cured	23	21	2	91.30	2.506	0.012
Recurrence	8	4	4	50.00		

Wilcoxon rank sum test (Z) or Kruskal-Wallis H test (Hc). OPG, osteoprotegerin; MGCs, multinucleated giant cells.

**Table II:** Correlation between expression of OPG in STCs and clinical pathology.

Category	No. of patients	OPG expression in STCs			Z or Hc	P-value
		Positive	Negative	Rate (%)		
Gender						
Male	18	13	5	72.22	0.290	0.828
Female	13	10	3	76.92		
Age (years)						
≤20	4	2	2	50.00	2.700	0.259
21–40	19	16	3	84.21		
>40	8	5	3	62.50		
Tumor site						
Surrounding knee joint	18	14	4	77.78	1.880	0.060
Other	13	9	4	69.23		
Jaffe’s grading						
I	12	9	3	75.00	0.789	0.674
II	17	12	5	70.59		
III	2	2	0	100.00		
Campanicci’s grading						
I	7	4	3	57.14	1.750	0.417
II	16	12	4	75.00		
III	8	7	1	87.50		
Prognosis						
Cured	23	19	4	82.61	1.786	0.074
Recurrence	8	4	4	50.00		

Wilcoxon rank sum test (Z) or Kruskal-Wallis H test (Hc). OPG, osteoprotegerin; STCs, stromal cells.

**Table III:** Correlation between expression of OPGL in MGCs and clinical pathology.

Category	No. of patients	OPG expression in MGCs			Z or Hc	P-value
		Positive	Negative	Rate (%)		
Gender						
Male	18	9	9	50.00	1.053	0.292
Female	13	4	9	30.76		
Age (years)						
≤20	4	4	0	100.00	6.232	0.044
21–40	19	6	13	31.58		
>40	8	3	5	37.50		
Tumor site						
Surrounding knee joint	18	7	11	38.89	0.398	0.691
Other	13	6	7	46.15		
Jaffe’s grading						
I	12	4	8	33.33	3.536	0.171
II	17	9	8	52.94		
III	2	0	2	0		
Campanicci’s grading						
I	7	2	5	28.57	4.699	0.095
II	16	5	11	31.25		
III	8	6	2	75.00		
Prognosis						
Cured	23	7	16	30.43	2.165	0.030
Recurrence	8	6	2	75.00		

Wilcoxon rank sum test (Z) or Kruskal-Wallis H test (Hc). OPGL, osteoprotegerin ligand; MGCs, multinucleated giant cells.

**Table IV:** Correlation between expression of OPGL in STCs and clinical pathology.

Category	No. of patients	OPG expression in STCs			Z or Hc	P-value
		Positive	Negative	Rate (%)		
Gender						
Male	18	11	7	61.11	0.914	0.361
Female	13	10	3	76.92		
Age (years)						
≤20	4	1	3	25.00	6.633	0.036
21–40	19	16	3	84.21		
>40	8	4	4	50.00		
Tumor site						
Surrounding knee joint	18	14	4	77.78	1.384	0.166
Other	13	7	6	53.85		
Jaffe’s grading						
I	12	11	1	91.67	7.705	0.021
II	17	10	7	58.82		
III	2	0	2	0		
Campanicci’s grading						
I	7	5	2	71.43	4.575	0.102
II	16	13	3	81.25		
III	8	3	5	37.50		
Prognosis						
Cured	23	15	8	65.22	0.502	0.616
Recurrence	8	6	2	75.00		

Wilcoxon rank sum test (Z) or Kruskal-Wallis H test (Hc). OPGL, osteoprotegerin ligand; STCs, stromal cells.
